# Regulation of phosphate transport and AMPK signal pathway by lower dietary phosphorus of broilers

**DOI:** 10.18632/oncotarget.22609

**Published:** 2017-11-22

**Authors:** Zhiqiang Miao, Yan Feng, Junzhen Zhang, Wenxia Tian, Jianhui Li, Yu Yang

**Affiliations:** ^1^ College of Animal Science and Veterinary Medicine, Shanxi Agricultural University, Taigu, Shanxi 030801, China

**Keywords:** AMPK signal pathway, broilers, lower dietary phosphorus, phosphate transport regulation

## Abstract

Lower available P (aP) was used as a base value in nutritional strategies for mitigating P pollution by animal excreta. We hypothesized that the mechanism regulating phosphate transport under low dietary P might be related with the AMPK signal pathway. A total of 144 one-day-old Arbor Acres Plus broilers were randomly allocated to control (HP) or trial (LP) diets, containing 0.45 and 0.23% aP, respectively. Growth performance, blood, intestinal, and renal samples were tested in 21-day-old broilers. Results shown that LP decreased body weight gain and feed intake. Higher serum Ca and fructose, but lower serum P and insulin were detected in LP-fed broilers. NaPi-IIb mRNA expression in intestine and NaPi-IIa mRNA expression in kidney were higher in the LP group. AMP: ATP, p-AMPK: total AMPK, and p-ACC: total ACC ratios in the duodenal mucosa were decreased in the LP group, whereas the p-mTOR: total mTOR ratio increased. These findings suggested that the increase in phosphate transport owing to LP diet might be regulated either directly by higher mTOR activity or indirectly by the suppressive AMPK signal, with corresponding changes in blood insulin and fructose content. A novel viewpoint on the regulatory mechanism underlying phosphate transport under low dietary P conditions was revealed, which might provide theoretical guidelines for reducing P pollution by means of nutritional regulation.

## INTRODUCTION

Phosphorus (P) is an essential nutrient for skeletal development in rapidly growing bird. However, there is increasing concern over excess P pollution by animal excreta in animal production systems. In addition, inorganic phosphates are a nonrenewable natural resources and its conservation is a global concern [1]. Consequently, the efficiency of P utilization has been focused on in contemporary research.

To address this issue, poultry nutritionists have developed several nutritional strategies, including the estimation of precise P requirements [2], dietary supplementation with feed additives, such as microbial phytase [3], vitamin D_3_ metabolites [4], and organic acids [5], as well as feed ingredients with low phytin-P [6, 7]. Most of the effective nutritional strategies set at lowering dietary available P (aP). Providing lower amounts of P than the estimated requirement was an effective method of reducing the excretion of P, and it was independent of phytase supplementation [8]. Another study showed that higher adaptive capacity was found in modern broilers subjected to early dietary P restrictions [9].

Dietary P restriction up-regulates the transport of Na^+^-dependent phosphate. Reducing dietary aP concentration by 43% stimulates Na^+^-dependent phosphate uptake and expression of the type IIb sodium-phosphate cotransporter (NaPi-IIb) protein in the brush border membrane of the small intestine [10]. NaPi-IIb is a critical transport protein for phosphate uptake in the small intestine, particularly in the duodenum [11]. However, reports on the mechanism underlying the regulation of intestinal absorption by low P are scarce.

AMP-activated protein kinase (AMPK), which is a sensor of peripheral energy balance, can be phosphorylated and activated by metabolic changes and an increase in the AMP:ATP ratio [12]. Once activated, AMPK switches off ATP-consuming biosynthetic pathways and switches on ATP-generating metabolic pathways. Feeding behavior is regulated by AMPK phosphorylation in the hypothalamus [13]. In addition, NaPi-IIb gene expression in the intestine is linked to the metabolic state of cells through AMPK activity, and AMPK has an inhibitory effect on NaPi-IIb in rats [14]. However, no data are available on the effect of dietary P on AMPK during the process of phosphate absorption in broilers.

In the present study, our aim was to reveal the mechanism regulating phosphate transport affected by low dietary P, from a novel viewpoint. We examined the effects of reduced dietary aP on blood parameters, phosphate transport mRNA expression in intestine and kidney, and the AMPK signal pathway protein expression in growing broilers. A certain relationship might be existed between phosphorus absorption and AMPK signal pathway by low dietary P.

## RESULTS AND DISCUSSION

### Effect of diets on growth performance of broilers

BWG and FI of broilers were significantly lower in the 0.23% aP diet group than in the control group (Table 1, *P* < 0.001). However, FCR was not different between the low and the control P groups, consistent with the findings of previous studies [15, 16]. The negative effect of feeding the diet with 0.23% aP on growth performance might have been due to a wider Ca:aP ratio (4.3:1), and the amount of aP in the diet [15]. A wider Ca:aP ratio could disturb P metabolism, thereby resulting in reduced growth, and this effect is more severe when dietary P level is too low [17]. In an earlier study, dietary P deficiency could suppress the Ca-specific appetite and growth of broilers [18].

**Table 1:** The influence of low aP levels on growth performance of broilers (*n* = 6)

aP^1^	BWG (g)^2^	FI (g)^3^	FCR^4^
LP^5^	560.15^A^	623.47^A^	1.21^b^
HP^6^	846.53^B^	947.36^B^	1.17^a^
SEM	4.52	5.76	0.006
*P*-value	< 0.001	< 0.001	0.08

^a-b^Within a column, values not sharing a common superscript letter are significantly different at 0.01 < *P* < 0.05

^A-B^Within a column, values not sharing a common superscript letter are significantly different at *P* < 0.01

^1^aP: available phosphorus

^2^BWG: body weight gain

^3^FI: feed intake

^4^FCR: feed conversion ratio

^5^LP: lower dietary phosphorus

^6^HP: higher dietary phosphorus.

Moreover, higher adaptive capacity was found in the modern broilers when they were subjected to early dietary P restriction (i.e., 0.30% aP as compared with 0.45% aP) [9], the body weight was lower in the initial phase, but could catch up with the control birds during the grower period, and had a higher apparent absorption of total P.

### Effect of diets on serum parameters of broilers

A study found that Ca:P ratios were related to the prevalence of central obesity [19]. We detected the relative index, and serum insulin was decreased in the group fed with lower P (Table 2, *P* < 0.05). Higher serum Ca and lower serum P were caused by low dietary P (*P* < 0.001). Insulin might be a regulator in phosphate absorption or re-absorption by ruminants [20] and a central regulatory factor in the control of nutrient partitioning during growth and development. A previous study on the relationship between growth and plasma insulin has shown that muscle protein synthesis was stimulated by insulin in growing animals [21]. Therefore, the lower body weight of broilers fed low dietary P might be related with the lower secretion of insulin.

**Table 2:** The effect of low aP levels on blood parameters (*n* = 6)

aP^1^	Ca (mg mL^−1^)	P (mg mL^−1^)	Insulin (IU L^−1^)	Fructose (mg mL^−1^)	Glucose (nmol L^−1^)
LP^2^	2.67^B^	1.155^A^	10.86^a^	7.26^b^	13.81
HP^3^	1.97^A^	1.808^B^	20.32^b^	6.36^a^	13.86
SEM	0.03	0.023	2.33	0.128	0.083
*p*-value	< 0.001	< 0.001	0.032	0.011	0.734

^a-b^Within a column, values not sharing a common superscript letter are significantly different at 0.01 < *P* < 0.05

^A-B^Within a column, values not sharing a common superscript letter are significantly different at *P* < 0.01.

^1^aP: available phosphorus

^2^LP: lower dietary phosphorus

^3^HP: higher dietary phosphorus.

Further we found that fructose in serum was higher in the group fed with low P, whereas the glucose concentration remained unchanged (*P* < 0.05). Low dietary P was reported to weaken insulin signaling through higher fructose concentration in human blood [22]. Excess consumption of fructose in humans increased body weight and induced adiposity, and hyperlipidemia in humans and rodents [23, 24]. Therefore, low-P feed might interfere with fat metabolism.

### Effect of diets on P transporter mRNA expression in duodenum and kidney

In the present study, NaPi-IIb mRNA expression in intestine and NaPi-IIa mRNA expression in kidney were both enhanced by low dietary P as compared with adequate P (Table 3). During P balance, P absorption and re-absorption take place in the intestine and kidney mainly through NaPi-IIb and NaPi-IIa. Up-regulation of small intestinal absorption of phosphate due to low dietary P was described in many species; the adaptive response to low-P diet leads to an increased kinetic parameters (Vmax) of Na-Pi cotransporter [25].

**Table 3:** The effect of low aP levels on NaPi-IIb and NaPi-IIa mRNA expression (*n* = 6)

aP^1^	NaPi-IIb mRNA expression in Duodenum	NaPi-IIa mRNA expression in Kidney
LP^2^	19.65^a^	3.45^B^
HP^3^	12.30^b^	2.18^A^
SEM	0.502	0.23
*P*-value	0.034	0.003

^a-b^Within a column, values not sharing a common superscript letter are significantly different at 0.01 < *P* < 0.05

^A-B^Within a column, values not sharing a common superscript letter are significantly different at *P* < 0.01

^1^aP: available phosphorus

^2^LP: lower dietary phosphorus

^3^HP: higher dietary phosphorus.

Dietary P deprivation could increase the serum 1,25-(OH)_2_D_3_ levels and enhance the 25-hydroxyvitamin D_3_ 1α-hydroxylase (1α-hydroxylase) protein and mRNA expression in kidneys [15, 26]. The synthesis of active vitamin D_3_ can be promoted by parathyroid hormone (PTH) through stimulation of the gene promoter 25-OH-D_3_-1α-hydroxylase [27]. PTH promotes Ca re-absorption and suppresses phosphate reabsorption in the proximal tubules [28]. In addition, dietary P regulation of 1,25 (OH)_2_D production was proved to be mediated by changes in circulating fibroblast growth factor 23 (FGF-23) [29]. FGF-23, a phosphatonin, regulates phosphate reabsorption in the kidney, and therefore plays an essential role in phosphate balance [30]. Moreover, FGF23 inhibits Na^+^-dependent inorganic phosphate transport, intestinal Na^+^-dependent P transport activity, and NaPi-IIb protein levels by a mechanism that is dependent on Vitamin D receptor [31]. FGF-23 could suppress the production of 1,25(OH)_2_D_3_, and decrease the protein expression of NaPi-IIa [32]. Serum FGF-23 concentration decreased significantly during dietary P restriction [30]; therefore, the inhibiting effect on expression of NaPi-IIa and NaPi-IIb protein by FGF-23 was weaken.

These results suggest that, during the earlier growth stage with P-restricted diet, P absorption and re-absorption were both enhanced in order to maintain an optimal level of P in the body. Bar *et al*. (2003) concluded that modern broilers exhibit high adaptability to P deficiency [33]. However, the strength was not enough to maintain growth.

### Effect of low dietary P on AMPK signal regulation

Previous studies have shown that high Ca and Vitamin D_3_ intakes caused lesser adiposity and lipid accumulation, probably via an insulin and AMPK- independent pathway [34]. However, few studies have reported about the effect of dietary P on the AMPK pathway. In the present study, AMPK phosphorylation and ACC phosphorylation in the intestine showed that p-AMPK:total AMPK ratio and p-ACC:total ACC ratio were decreased, and p-mTOR:total mTOR ratio was increased in the intestine of broilers feeding on a low-P diet (*P* < 0.05, Table 4). The novel and important findings of the present study are that the low-P diet resulted in decreased AMP:ATP ratio in the duodenum of chickens, which was positively correlated with AMPK phosphorylation. NaPi-IIb gene expression is linked to the metabolic state of cells through AMPK activity, whereas NaPi-IIb in the small intestine might be inhibited by AMPK activation in rats [14]. Furthermore, mTOR, which is a kinase known to regulate many intestinal nutrient transporters, is inhibited by the activation of AMPK [35]. In addition, NaPi-IIb has been shown to be stimulated directly by mTOR [36]. Therefore, in the present study the higher NaPi-IIb mRNA expression induced by low dietary P might be stimulated directly by mTOR or indirectly by AMPK signals.

**Table 4:** The influence of low aP levels on AMPK signal pathway (*n* = 6)

aP^1^	AMP:ATP ratio	p-AMPK:T-AMPK	p-ACC:T-ACC	p-mTOR:T-mTOR
LP^2^	59.85^A^	0.643^a^	0.587^a^	0.482^b^
HP^3^	76.85^B^	0.906^b^	0.729^b^	0.429^a^
SEM	5.51	0.06	0.036	0.01
*P*-value	< 0.001	0.03	0.017	0.024

^a-b^Within a column, values not sharing a common superscript letter are significantly different at 0.01 < *P* < 0.05

^A-B^Within a column, values not sharing a common superscript letter are significantly different at *P* < 0.01.

^1^aP: available phosphorus

^2^LP: lower dietary phosphorus

^3^HP: higher dietary phosphorus.

ACC is a downstream target protein of the AMPK signal pathway. Phosphorylated AMPK could inhibit the activity of ACC by enhancing ACC phosphorylation, which decreases the synthesis of fatty acid. In the present study, ACC phosphorylation was decreased and its activity was enhanced, accompanied by lower AMPK phosphorylation. It implies that the synthesis of fatty acids would be increased correspondingly, and this process is an ATP-consuming biosynthetic pathway. This might be the reason underlying the low P status, which is reported to contribute to the onset of obesity [37]. Conversely, HP diet was suggested to enhance energy expenditure through the utilization of free fatty acids, which were released via lipolysis of white adipose tissue, and decrease lipid accumulation [38, 39]. This was consistent with the higher fructose concentration in blood induced by low-P diet, which might induce adiposity [24].

In conclusion, low dietary P increased the NaPi-IIb mRNA expression in the intestines and NaPi-IIa mRNA expression in the kidneys, in addition to lower blood insulin and higher fructose levels. This process might be regulated directly by mTOR or indirectly through AMPK signal. The present study revealed the regulatory mechanism of phosphate transport affected by low dietary P from a novel viewpoint. This might provide theoretical guidelines for reducing P emission by means of nutritional regulation.

## MATERIALS AND METHODS

### Animals and diets

The present study was performed in accordance with the Guidelines for Experimental Animal Welfare of the Ministry of Science and Technology of China (Beijing, P. R. China), after approval. A total of 144 one-day-old Arbor Acres Plus broilers were purchased from a local hatchery and randomly allocated to 12 pens, with 12 chickens in each pen.

The chickens were fed with diets containing either 0.45% (high) aP as a control, or 0.23% (low) aP as a trial group. Each of the two diets was fed to six replicate pens. The other nutrients were based on the recommendations of the National Research Council in 1994. The dietary composition and nutrient content are shown in Table 5.

**Table 5:** Composition of diets and nutrient levels in broilers

	0-21 days	
	Trial diet (LP)	Control diet (HP)
**Ingredients (%)**		
Corn	41.44	41.44
Soybean meal	44.72	44.72
Soybean oil	9.36	9.36
Limestone	2.2	1.32
Dicalcium phosphate	0.26	1.64
Maifanite	1	0.5
DL-methionine	0.16	0.16
Salt	0.30	0.30
Trace mineral premix^1^	0.20	0.20
Vitamin premix^2^	0.03	0.03
50% Choline chloride	0.30	0.30
Antioxidant	0.03	0.03
Total	100	100
**Nutrient composition**		
ME (MJ kg^-1^)	13.37	13.37
CP^3^ (%)	23.00	23.00
Ca (%)	1.00	1.00
Available P^4^ (%)	0.23	0.45
Total P^4^ (%)	0.43	0.65
Lysine (%)	1.20	1.20
Methionine (%)	0.52	0.52
Tryptophan (%)	0.33	0.33
Threonine (%)	0.97	0.97

^1^Nutrients per kilogram of diet: Cu (from CuSO_4_·5H_2_O), 16 mg; Fe (from FeSO_4_·7H_2_O), 80 mg; Zn (from ZnSO_4_·7H_2_O), 110 mg; Mn (from MnSO_4_·H_2_O), 120 mg; I (from Ca(IO_3_)_2_·H_2_O), 1.5 mg; Co (from CoCl_2_·6H_2_O), 0.5 mg; Se (from organic selenium), 0.3 mg.

^2^Nutrients per kilogram of diet: vitamin A, 12,500 IU; vitamin D_3_, 3,000 IU; vitamin E, 25 mg; vitamin K_3_, 2.5 mg; thiamin, 2.5 mg; riboflavin, 8 mg; vitamin B_12_, 0.025 mg; folic acid, 1.25 mg; niacin, 37.5 mg; pantothenic acid, 12.5 mg; biotin, 0.125 mg.

^3^Crude protein (CP) content of corn is 8.7%; CP content of soybean meal is 43%.

^4^The analyzed levels of available P were 0.22 %, 0.46 %. The analyzed levels of total P were 0.45%, 0.67%.

The chickens were reared under controlled environments. The temperature was initially maintained at 34°C when the broilers were 1-3 days old, followed by a gradual decrease to room temperature (24°C). The light regimen was 23:1 (light: dark). All the birds were provided mashed feed and tap water *ad libitum*.

### Sample collection

At the age of 21 days, the body weight of each broiler was measured 4 h after feed withdrawal and the feed remaining in the trough was weighed. Body weight gain (BWG), feed intake (FI), and feed conversion ratio (FCR) from days 1-21 was calculated.

After the birds were weighed, blood sample was drawn from a wing vein of one bird from each pen, weighing closest to the mean body weight for each treatment. Serum was obtained after centrifugation at 3,000 × *g* for 10 min and was stored at -20°C for further analysis. Immediately after the blood sample was obtained, the chickens were killed by cervical dislocation. 10-cm segments of duodenal tissues of these 21-day-old broilers were cut longitudinally, and the contents were flushed with ice-cold PBS. Mucosal samples were rapidly collected by scraping with a sterile glass microscope slide, immediately frozen in liquid nitrogen, and stored at −80°C until analysis.

### Blood parameters

The concentration of glucose (No. F006), fructose (No. A085), Ca (No. C004-1) and P (No. C006) in serum were measured spectrophotometrically using commercial diagnostic kits (Jiancheng, Nanjing, China). Serum insulin level was measured by radioimmunoassay as Liu *et al.* (2012) described [40].

### Determination of mucosal ATP and AMP content

Frozen mucosal samples (100-200 mg) were homogenized with 2 mL pre-cooled 1.5 mmol/L sodium fluoride-perchloric acid in an ice-bath [41]. The homogenates were centrifuged at 3, 000 ×*g* for 10 min at 4°C. One milliliter of the supernatant was neutralized with 0.4 mL of 2 M potassium carbonate on ice, and the solution was centrifuged at 3, 000 ×*g* for 5 min at 4°C. The ATP and AMP content was analyzed according to the method of Hou *et al*. (2011) using high-performance liquid chromatography [42].

### Total RNA extraction, reverse transcription, and real-time PCR

Total RNA was extracted from the duodenal mucosa of the 21-day-old broilers using the SV Total RNA Isolation System Kit (Z3100; Promega, Madison, WI, USA), according to the manufacturer's instructions. The RNA was re-suspended in diethyl pyrocarbonate-treated water. The concentration and quality of the RNA were determined by measuring the absorbance at 260 nm and agarose gel electrophoresis, respectively.

After extraction, 1.0 μg total RNA was used as the template for synthesizing single-stranded cDNA with avian myeloblastosis virus reverse transcriptase (Promega, Madison, WI, USA) using a 15-mer oligo (dT) primer in the presence of recombinant RNasin ribonuclease inhibitor (A3500; Promega, Madison, WI, USA).

Real-time PCR of NaPi-IIb, NaPi-IIa were performed with β-actin as the internal control. The primers and amplicon sizes are presented in Table 6. Real-time PCR was conducted in an ABI 7500 Fluorescent Quantitative PCR system (Applied Biosystems, Bedford, MA) using the RealSuper mixture with Rox (CW0767; CWbio Company, Sandringham, UK). The PCR conditions were as follows: 95°C for 4 min, followed by 40 cycles of 95°C for 15 s and 60°C for 60 s, and 60–95°C for melting curve analysis. Each gene was amplified in triplicate. Standard curves were analyzed simultaneously to determine the efficiency of amplification. The results were expressed as the ratio of the target gene mRNA to β-actin mRNA, and the differences in gene expression were determined using the cycle threshold method [15].

**Table 6:** Oligonucleotide PCR primers

Gene	GenBank accession	Orientation	Primer sequence (5ʹ→3ʹ)	Predicted size (bp)
NaPi-IIb	NM_204474.1	Forward	CTTTTACTTGGCTGGCTGGAT	148
		Reverse	AGGGTGAGGGGATAAGAACG	
NaPi-IIa	AF297188.1	Forward	CCGCACCTCCCCAGACT	100
		Reverse	GTTGTGGAGGATCCCAATGC	
β-Actin	NM_205518.1	Forward	AACACCCACACCCCTGTGAT	100
		Reverse	TGAGTCAAGCGCCAAAAGAA	

### Western blot analysis

The duodenal brush-border membrane vesicles were homogenized and centrifuged at 10,000 ×*g* for 5 min at 4°C. The resulting supernatants were stored at −80°C until further use. Protein concentration was determined by the Bradford assay. The samples of brush-border membrane vesicles were placed in Laemmli buffer (Sigma-Aldrich, St. Louis, MO, USA) and boiled for 5 min to induce protein denaturation. Thereafter, 50 mg of brush-border membrane vesicle proteins were loaded onto each lane and electrophoresed on a 4% polyacrylamide gel. The proteins were subsequently transferred onto a polyvinylidene difluoride membrane for 2 h, followed by probing the polyvinylidene difluoride membrane for the presence of target proteins by incubation with the following primary antibodies (AMPK-α antibody, P-AMPKα (Thr172) antibody, ACC (Acetyl-CoA carboxylase) antibody, P- ACC antibody, mTOR antibody, P-mTOR(Ser2448 antibody), diluted to 1:1000, for at least 1 h. The antibodies were purchased from Cell Signaling Technology (Inc., Beverly, MA, USA). The membrane was washed in Tris-buffered saline Tween-20 and incubated with a secondary antibody conjugated with horseradish peroxidase (1:5000; Bio-Rad, Hercules, CA, USA). The immunoblots were visualized on an X-ray film through chemiluminescence (Pierce Protein Research Products, Rockford, IL, USA), and optical density-calibrated images were analyzed using AlphaEase stand-alone software (Alpha Innotech, Santa Clara, CA, USA) [43].

### Statistical analyses

The results were analyzed by one-way analysis of variance (ANOVA) using SPSS ver. 17.0 (IBM-SPSS, Inc., Chicago, IL, USA). The replicate means were used as experimental units in this analysis. When a treatment was significant (*P* < 0.05), the differences between the means were assessed using Tukey's honest significant difference multiple range analysis. Prior to analysis, the homogeneity of variance was examined, and the normality of data was verified. Data are presented as mean values.
